# Supplementary material for: A multivalent binding model infers antibody Fc species from systems serology
Source: PLoS Comput Biol. 2024 Dec 23;20(12):e1012663. doi: 10.1371/journal.pcbi.1012663 (PMC11706497; doi:10.1371/journal.pcbi.1012663)
Supplement: S1 Text — Reasoning behind chosen distributions of synthetic Fc species abundances. (DOCX) [file pcbi.1012663.s001.docx]

## S1 Text. Generating synthetic Fc species abundances

The real distributions of antibody Fc species across samples and antigens are difficult to determine directly. To decide on a distribution from which to sample Fc species abundances in our synthetic tests, we instead relied on the distributions of anti-subclass detections from real systems serology datasets. These measurements depend on the abundance of their target subclass in a way that does not depend on the abundance of other subclasses, and so they are somewhat representative of the underlying Fc species abundances. However, an important limitation of these subclass-specific detections, as we have mentioned, is that their signal depends nonlinearly on the abundance of their target species. Despite this limitation and because of the scarcity of reasonable alternatives, we used the distributions of these measurements to design the distribution of species abundances in our synthetic tests; specifically, we replicated the log-normal distributions that we often saw in these measurements and chose a relative mean and standard deviation for our synthetic distribution to resemble the measurements (S1 Fig).
