# Supplementary material for: A multivalent binding model infers antibody Fc species from systems serology
Source: PLoS Comput Biol. 2024 Dec 23;20(12):e1012663. doi: 10.1371/journal.pcbi.1012663 (PMC11706497; doi:10.1371/journal.pcbi.1012663)
Supplement: S2 Text — Comparison of fucosylation inferences from the binding model with fucosylation measurements from capillary electrophoresis. (DOCX) [file pcbi.1012663.s002.docx]

## S2 Text. Comparison of fucosylation inferences from the binding model with fucosylation measurements from capillary electrophoresis

We compared our fucosylation inferences to direct measurements of IgG fucosylation. While direct measurement of antibody glycosylation is generally more challenging, in one study these measurements are available along with the detection binding data. In this study, by Alter et al, detection binding data was collected for serum samples from a cohort of HIV patients using a panel of HIV antigens [1]. In parallel, the authors chose one HIV antigen, gp120.SF162, to capture antibodies from each subject, and used capillary electrophoresis (CE) to measure the percentage of these antigen-specific antibodies that possessed certain glycans. This dataset, including joint detection binding and fucosylation data, presented a unique opportunity to validate the model’s inferences directly. We used these CE measurements to validate our model’s inferences by using the binding model to infer the quantity of each Fc species targeting gp120.SF162 and comparing the fraction of fucosylated antibodies among these inferences to that measured with CE (S5a Fig). There is a weak agreement between the raw CE measurements and the model’s inferences (S5b Fig).

However, the CE glycosylation measurements contradict both the FcR binding data and effector function measurements, and thus are likely inaccurate. While there is no way to directly validate the CE measurements, we compared them to other measurements on the same IgG fraction to see if they are consistent with existing mechanistic understanding. IgG fucosylation blocks binding to FcγRIIIA, with little to no effect on the binding to α-hIgG1 IgG [2] and FcγRIIA. Therefore, we expect IgG fucosylation to be negatively correlated with the ratios FcγRIIIA / α-hIgG1 and FcγRIIIA / FcγRIIA. However, no such relationships exist for the CE fucosylation measurements (S5c and S5d Fig), while they do exist for the binding model fucosylation inferences (S5e and S5f Fig). The difference between the CE fucosylation measurements and the detection signals could arise from inaccuracy in the former, the latter, or both. If the detection signals are inaccurate, then we would also expect the binding model fucosylation inferences to be inaccurate as these inferences are computed from the detection signals.

To examine whether the disagreement between the detection signals and CE fucosylation arises from inaccuracy of the former or latter, we compared these measurements to one other measurement modality: effector function measurements. We see that FcγRIIIA binding is positively correlated with ADNKA (S5g Fig), which is expected because FcγRIIIA is an excitatory receptor expressed primarily on NK cells [3]. Additionally, we see that FcγRIIA is positively correlated with ADNP (S5h Fig.), which is expected because FcγRIIA is an excitatory receptor expressed on neutrophils [3]. Together, these results indicate that the effector function measurements and FcγRIIA, FcγRIIIA signals are consistent with each other.

Interestingly, the CE glycosylation measurements are consistent with themselves. The presence of bisecting GlcNAc on the N-glycan of IgG1 inhibits the addition of a fucose residue [4,5]. This mechanism is reflected in the negative correlation between CE-measured IgG fucosylation and CE-measured IgG bisecting GlcNAc (S5i Fig). This relationship is inconsistent with any theory that proposes faults in individual CE features. Alternatively, one explanation for the CE measurements contradicting the detection binding and effector measurements is a difference in analyzed antibody population between the former and latter two methods. The detection binding and effector measurements were both collected from gp120-bound IgG (a bead in the former case and a target cell in the latter case), while the CE measurements were collected from IgG which bound to gp120 and were subsequently eluted with low pH [1], with possible effects on the isolated antibody population.

# **References**

1. Alter G, Dowell KG, Brown EP, Suscovich TJ, Mikhailova A, Mahan AE, et al. High‐resolution definition of humoral immune response correlates of effective immunity against HIV. Mol Syst Biol. 2018 Mar;14(3):e7881.

2. Šuštić T, Van Coillie J, Larsen MD, Derksen NIL, Szittner Z, Nouta J, et al. Immunoassay for quantification of antigen-specific IgG fucosylation. eBioMedicine. 2022 Jul;81:104109.

3. Bruhns P, Jönsson F. Mouse and human FcR effector functions. Immunol Rev. 2015 Nov;268(1):25–51.

4. Golay J, Andrea AE, Cattaneo I. Role of Fc Core Fucosylation in the Effector Function of IgG1 Antibodies. Front Immunol. 2022 Jun 30;13:929895.

5. Nakano M, Mishra SK, Tokoro Y, Sato K, Nakajima K, Yamaguchi Y, et al. Bisecting GlcNAc Is a General Suppressor of Terminal Modification of N-glycan*[S]. Mol Cell Proteomics. 2019 Oct;18(10):2044–57.
