# Supplementary material for: A multivalent binding model infers antibody Fc species from systems serology
Source: PLoS Comput Biol. 2024 Dec 23;20(12):e1012663. doi: 10.1371/journal.pcbi.1012663 (PMC11706497; doi:10.1371/journal.pcbi.1012663)
Supplement: Attachment — Submitted filename: response to reviews.pdf [file pcbi.1012663.s008.pdf]

Response to reviews

We thank the reviewers for their thoughtful and constructive suggestions to strengthen this work. Their insights on improving our model's validation, enhancing the clarity of our presentation, and refining the focus of our manuscript are greatly appreciated. We are committed to addressing each point raised and are confident that these revisions significantly enhance the impact and accessibility of our research.

Reviewer #1: Summary: This work addressed a novel computational model application to provide a more accurate proxy for quantifying antibody abundance and properties. This challenge has been extensively explored in omics research and demonstrated significant potential for application in antibody response profiling, supporting immunological discoveries and vaccine design. By detailing their binding model and validating its implementations with existing antibody profile datasets, the authors highlighted several advantages of their approach, including simplifying serological protocols and advancing the new discovery including the understanding of the relationship between antibody structure and vaccine efficacy.

Major:

1. While inferring true antibody abundance is challenging, the impact of this work could be significantly enhanced by elaborating on existing benchmarks and providing additional performance comparisons beyond the use of raw measurement inputs.

Designing benchmarks is difficult in this scenario, as the binding model is providing information that is not otherwise directly available from systems serology. Therefore, its advantage is its provision of entire new avenues of inquiry. Despite the limitations of benchmarks in this context, we decided to add one benchmark: predicting effector function from inferred antibody Fc species. These results are in the newly added figures 4f and 4g.

2. The authors utilized synthetic data simulation and incorporated Gaussian-distributed noise to demonstrate their method's ability to reconstruct the "ground truth" antibody quantity. However, in practical applications, distinguishing between noise and a genuine lack of signal can be challenging. The manuscript would benefit from a discussion on the implications of noise removal for false positive/negative discoveries or from additional simulations that address real-world data complexities.

This test was not intended to evaluate the capability of our model to remove noise, but rather to evaluate whether the model performs acceptably in the presence of noise, where "acceptable" does not entail removing the noise that was added. One concrete scenario we wanted to rule out was that in which the model amplifies the noise in the measurements in producing its outputs. We have clarified this on lines 220–222.

3. In the introduction, the authors have highlighted the use and development of advanced statistical and machine learning methods in systems serology studies. To enhance the

comprehensiveness of this work, it would be beneficial to integrate a discussion on mathematical modeling methods, especially in the context of advancing or comparing these techniques with machine learning applications in immunological profile studies in tasks including signal annotation and missing value imputation.

We assume by "mathematical modeling methods" you mean "mechanistic modeling methods". We had previously mentioned another mechanistic modeling study in the introduction, but we did not properly contextualize this study by placing it in the paragraph with the other computational modeling approaches for systems serology. We added some analysis of this study in the paragraph on other modeling approaches on lines 82–89 and reworded our subsequent reference to it in the paragraph where we talk about Fc structural variations on lines 105–107.

Minor:
1. Fig 3. c, Fig.7.e-h were labeled with both r and R2. Please explicit what these are in the figure legends or labels for better information delivery.

This has been modified in the figure labels for figures 3 and 7.

2. The color scheme in Figure 5 used the same sets of colors for different underlying subgroups; Panel a and c used the same set of x-axis but are ordered differently.

New colors were chosen and the x-axes were reordered.

3. An overall color legend for Fig 7. a-d is helpful for visualization.

A color legend was added to figure 7.

Reviewer #2: The SARS-CoV-2 vaccine produces a spike antigen to induce an immune response. Abraham et al used computational techniques to find that the efficacy of this vaccine is correlated with the raising of an immune response towards the spike antigen. They also examined HIV. Though it may appear obvious that an immune response must be raised for the vaccine to be efficacious, the authors aim to unveil mechanistic details of this process. The manuscript appears to be an amalgamation of several interesting, but only loosely connected, research tidbits - the readability of future work would be improved with a tighter focus, even if it means a smaller paper. This would also help readers better understand how it applies to their own work, and how they could use it to advance their own work. The admission of shortcomings, and descriptions of small failures (i.e. "At first, we attempted to fit γ, but this made the fitting too flexible, resulting in unrealistic values of 515" and paragraphs such as that one), is a strength of this work, as these bits of knowledge can speed up future research progress for others. Overall, this was an interesting manuscript to read, and the authors did a thorough job with their work. After making some revisions for manuscript clarity, I believe this work should be published in PLoS Computational Biology.

1) The authors mention that they need to estimate the crosslinking constant from a previous

estimate. Building a computational model to infer experimental data is tricky to begin with, and building this model based off of estimates of experimental data is moving towards shaky ground. How confident are the authors that this is a reasonable estimate? What can they do to show naive readers that this estimate should be trusted?

We thank the reviewer for pointing out this crucial detail. Due to experimental data constraints, it is quite difficult to verify our Kx* estimate. However, we can investigate how much the model's performance is impacted by inaccuracies in the Kx* estimate using our synthetic validation pipeline. We have added a paragraph describing this on lines 237–242 and we have added figure S2.

2) There are several instances, such as in the abstract and introduction, where the authors imply that they are studying broad mechanistic details of antibody binding and the raising of an immune response, while they actually have a narrow focus of inferring glycosylation patterns of antibodies and deducing which experimental systems serology data points are necessary, and which are unnecessary, in antibody assays. This is valuable in its own right, and the manuscript would be strengthened by the authors narrowing their claims. The current broader focus of the manuscript dilutes and muddies the worthwhile results that the authors have found, and would make it more difficult for an average reader to understand this work. Furthermore, from the results section it appears that these results are only applicable to the SARS-CoV-2 and HIV vaccines, whereas the abstract and introduction imply that the authors used these two vaccines to make a model that can draw broader conclusions about immune responses.

We thank the reviewer for bringing this concern to light, and we see how this interpretation arose. We think that this comment points out two important issues with the text: (1) we did not properly specify how general the model was and the concrete implementation details behind this generality, we merely claimed that it was general and (2) we did not justify why we focused on the applications we did, and this perhaps conveys that these applications were chosen because the model was limited to them. We address (1) by providing a brief addition to the description of the model's usage on lines 144–146 and we address (2) by prefacing the COVID-19 and HIV analysis with a justification for our choice of model application on lines 377–382.

3) "However, both FcγRIIa and FcγRIIb gain a significant bias in their predictions as the number of masked values is increased, as indicated by the increasing R2." Why is this the case? Why do some antibodies gain bias while others don't?

We have provided the explanation that we think is most likely on lines 287–293.

4) The model can infer fucosylation because of the reduction in binding affinity between IgG and FcγRIIIA. Is this fucosylation prediction generalizable to other pairs, such as IgG and FcγRIIA?

No, this binding affinity effect is limited to FcγRIIIA, and this is one of the reasons that the model can infer the quantities of fucosylated antibodies. We thank the reviewer for bringing this limitation of our explanation to our attention. We have more explicitly detailed the effects of

fucosylation and the importance of these effects in our binding model on line 308 and lines 319–324.

5) In one section, the authors mention that "This correlation between HIV infection severity and IgG afucosylation is like the correlation we found between COVID-19 severity and IgG afucosylation." Thus afucosylation can be predictive of COVID-19 severity and HIV infection severity, in those specific cases. However, the authors later state that "We found a high degree of variation in IgG fucosylation by antigen across subjects (Fig. 6d), indicating that antigen-specific IgG fucosylation cannot be inferred using the IgG fucosylation for another antigen from the same pathogen." These two statements appear contradictory. If there is a high degree of variation in IgG fucosylation, then it would appear not to be directly predictive of infection severity. It would be preferable to have a general model that can predict fucosylation regardless of the antigen in question, but is understandable that this is not the case. However, one would expect the conclusions to be the same regardless of the antigen in question (i.e. that afucosylation is predictive of infection severity). If this is not the case, an explanation should be provided for clarity.

This is an excellent observation. This is an important question and one we overlooked. While it is unclear for which antigens there is this negative correlation between IgG fucosylation and disease severity, there are some patterns we can identify. We added a new subplot, figure 6d, which plots the fucosylation differences across disease response, grouping the antigens by their structural domain. We have added analysis for this plot on lines 437–439 and a holistic discussion of these updated figures on line 535. In short, we say that the antigens against which IgG fucosylation is related to disease severity are a strict subset of antigens composing the Env trimer.

6) "This study includes CE IgG fucosylation measurements for the gp120.SF162 HIV antigen10" As written, it appears that the submitted manuscript contains CE IgG fucosylation measurements, and the citation above is a general citation for the gp120.SF162 HIV antigen. This should be rewritten for clarity.

We think this may be a misinterpretation of this sentence as "Our study includes CE IgG fucosylation measurements…". This data comes from another study, and we are citing this study. We have added a few words clarifying the source of this data when we introduce it, at line 410.

7) The source code is provided on GitHub. However, there is no README or instructions for users to either build the model for themselves, or for users to download prebuilt models. Additionally, it is unclear whether the authors expect users to build models for themselves or download prebuilt models. These should be remedied to improve the usability of the work.

We have improved the README file in the GitHub repository.

Reviewer #3: In this manuscript, Abraham and colleagues developed a model for inferring Fc structural features from systems serology assays. Having such a model is of great value as it would potentially reduce the need for lengthy wet-lab experimentation.

Please see my comments below.

Title: The term "antibody species" is somewhat unusual. At least to me, it was not clear that it would describe Fc features. Consider rewording.

We chose to use "antibody Fc species" or "Fc species" for short. This is reflected throughout the text and in each figure which uses this name.

Figure 1: it's not immediately clear from this figure, what the model's purpose and output is. Consider revising.

We added some more text to figure 1 to clarify both the model's purpose and output.

Figure 2: To what extent does the synthetic date recapitulate the key characteristics of experimental data? In other words, how biologically relevant are the simulations?

We have added supplemental figure S1 comparing the synthetic Fc species abundances to the subclass-specific detection measurements and referenced this figure on line 195.

Figure 3: what's the maximum amount of missing data (%) you can infer? What's the influence of dataset size on imputation capacity?

The maximum amount of missing data that the model can infer is 100% which is stated on line 285 and shown in figure 3g,h. We have also added supplementary figure S4, which shows the imputation performance at a fixed missingness ratio and variable dataset size and reference this figure on line 284.

Figure 4: Can you clarify whether these measurements are experimentally validated? If not, please add somewhat of a confidence measurement to your predictions (if possible) and maybe weaken the statements attached to this figure a bit.

Yes, the measurements are experimentally validated. We have modified line 342 to clarify this.

Figure 5: Please show the data points in all box plots (for all boxplots in the paper).

We have shown the data points in all box plots in the paper.

Model: Consider moving some of the mathematical details of model derivation to the main text. This would help the reader more quickly grasp the main variables of the model.

While such an approach would allow those with a mathematical background to more quickly comprehend our model, we want our paper to be accessible to immunologists without a mathematical background. We believe that leaving out mathematical equations from the main

text would make the text more accessible to this audience, and given the extensive mathematical description in the methods section, we think that our approach caters to both groups of readers.

Can the authors comment on the batch-to-batch variability of the model? In other words, are parameters learned from one dataset, applicable to another dataset where the biological sample was the same? Have the authors tried to apply the model to technical and biological replicates to understand whether the parameter inference is robust?

We thank the reviewer for bringing up this valuable consideration. Unfortunately, data for replicates are not readily available in the systems serology datasets we have used in this paper, thus hindering any validation approaches that rely on replicates. We do think that this issue is worth discussing in the manuscript. Perhaps unlike other modeling approaches, this approach is designed to infer almost exclusively concrete and physical quantities of the biological sample. This is to be differentiated from abstract characterizations generated by ML and statistical approaches that are commonly taken in current literature. This distinction is important because it is in no sense correct for our inferences to include any artifacts or batch effects, whereas it is quite understandable if the other ML and statistical approaches generate inferences that contain batch effects. It also means that a comparison of the model's inferences can be compared across datasets in a more straightforward way. There is a small subset of parameters in our model that are learned on a dataset-by-dataset basis, and thus will encapsulate batch effects. However, the idea is that the batch effects are constrained to this subset of parameters, and thus the Fc species inferences are left without batch effects. We have added a brief discussion of this starting on line 299.
